# Digging up food: excavation stone tool use by wild capuchin monkeys

**DOI:** 10.1038/s41598-017-06541-0

**Published:** 2017-07-24

**Authors:** Tiago Falótico, José O. Siqueira, Eduardo B. Ottoni

**Affiliations:** Dept. of Experimental Psychology – Institute of Psychology, University of São Paulo, São Paulo, SP Brazil

## Abstract

Capuchin monkeys at Serra da Capivara National Park (SCNP) usually forage on the ground for roots and fossorial arthropods, digging primarily with their hands but also using stone tools to loosen the soil and aid the digging process. Here we describe the stone tools used for digging by two groups of capuchins on SCNP. Both groups used tools while digging three main food resources: *Thiloa glaucocarpa* tubers, *Ocotea* sp roots, and trapdoor spiders. One explanation for the occurrence of tool use in primates is the “necessity hypothesis”, which states that the main function of tool use is to obtain fallback food. We tested for this, but only found a positive correlation between plant food availability and the frequency of stone tools’ use. Thus, our data do not support the fallback food hypothesis for the use of tools to access burrowed resources.

## Introduction

Capuchin monkeys (*Sapajus* spp and *Cebus* spp) use extractive foraging techniques – sometimes tool-aided - to obtain hard-to-acquire food, like larvae from tree branches/bark or encased seeds^1–5^; some *Sapajus* populations use probe tools to expel small prey, dip for honey and fish termites^6–8^. Another hard to obtain, concealed, food sometimes consumed by primates are plants’ Underground Storage Organs (USOs), usually rich in carbohydrates.

Fallback food is constantly proposed as an important selective force that could determine primate anatomy, influence grouping and ranging behavior, and trigger adaptation processes in primate evolution^9, 10^. The use of USOs as fallback food (a less preferred resource used during food shortage) is a selective force in some models on primate and human evolution^9, 11^, which state humans would have used this kind of resource during the shortage of food in dry seasons.

Fallback food classification has been proposed depending on the preferred food quality and the fallback food available^9^. The term “staple fallback foods” can be used to describe resources that are used as the only food in periods of low availability of preferred food. On the other hand, “filler fallback foods” can be defined as resources that do not encompass the entire diet at the low food period^12^. Usually low quality food (leaves, bark) requires more processing and anatomical adaptations, and high quality food (eg fruits and seeds) depend more on the behavioral adaptations for foraging the food^13^.

Until recently, it was thought chimpanzees could be a good model for the use of USOs as fallback food by primates, because they dig for the USOs of some plants, in some cases using tools^14–16^. This practice was interpreted as a fallback food strategy^11^. More detailed studies showed that it was not the case, since chimpanzees use this resource more frequently during the food-rich wet season^14^, thus not supporting the fallback strategy.

Capuchin monkeys (*Sapajus* spp and *Cebus* spp) are New World primates that have a large area of occurrence in Central and South America, inhabiting diverse environments, from rainforests to dry-bush forest, and semi-arid areas^17^. In tropical forests, they do not usually forage for roots or USOs^18, 19^, but there are reports of crop raid on cassava tubers in groups of *Sapajus nigritus* ^20^. More terrestrial populations, like *S*. *libidinosus* living in savannah environments such as *Caatinga*, may forage frequently on roots and USOs^7, 21, 22^.

The *S*. *libidinosus* population of Serra da Capivara National Park (SCNP) has the largest known tool kit for wild capuchin monkeys, including stone tools for foraging^1, 7, 21, 23^, threat^24^ and sexual displays^25^; and sticks as probing tools^6, 7, 26^. Moreover, capuchin monkeys at SCNP usually forage on the ground for various roots and fossorial arthropods, digging mainly with their hands but also using stone tools to loosen the soil and aid the digging process^7^.

One of the explanations for the innovation and maintenance of tool use is the “necessity hypothesis”, that states tool use is maintained by the need to access fallback food during times of preferred food scarcity, like dry seasons^27, 28^. Koops *et al*.^27^ tested the “necessity hypothesis” for tool-assisted insectivory in chimpanzees, but found that although the termites and ants caught with tools were present during times of scarcity, they were not used as fallback food, and nuts cracked with stone tools were not available during food shortage periods.

This hypothesis was tested for the use of pounding stones for nutcracking in another population of *S*. *libidinosus* (Fazenda Boa Vista) and was not supported^28^. In this same study, the “opportunity hypothesis” - tool use innovation sustained by repeated exposure to appropriate ecological conditions, like the availability of both food that requires tools for processing and potential tools - was also tested and supported.

Here we (1) describe the stone tools used to aid digging by two groups of capuchins in SCNP during two years, and the characteristics of the stone tools, (2) compare the data to observations from other groups living 15 km apart in the same park, by Mannu and Ottoni^7^ and Moura and Lee^21, 23^, and (3) test the “necessity hypothesis” for digging stone tools’ use. If this hypothesis is to be supported, we expect the frequency of tool use to be higher when the overall food availability is lower (eg. in the dry season). We used the monthly availability of arthropods and plant items (fruits/seeds/flowers) in the area as measures of food availability.

## Methods

### Study site

The research was conducted in Serra da Capivara National Park (Piauí State, northeastern Brazil). The park is located at the geoclimatic domain of the *Caatinga*, semi-arid climate with vegetation composed of a mosaic of xerophytic vegetation and patches of deciduous forest at narrow, wetter valleys surrounded by high cliffs. The annual rainfall is concentrated in the short wet season, from November to March. The study area was the Boqueirão da Pedra Furada, in the southeastern border of the park (8°50′S, 42°33′W).

### Study groups

We observed two not previously studied groups with overlapping living areas. At the beginning of the study, Pedra Furada (PF) group was composed of 45 individuals, and Bocão (BC) group had 27 individuals. The two groups sometimes met and foraged in the same area for minutes or hours, but no agonistic encounters were registered between them. Agonistic episodes occurred between individuals from the two groups, but did not include the whole groups. Capuchin monkeys at SCNP obtain most of their food by exploiting naturally occurring resources, but the park staff provisioned both groups during the dry season. The provisions consisted of fruits (3–4 times a week) and dry corn (every 2 weeks). The corn was meant for other animals in the park, but the monkeys also cosumed it.

### Observation Method

The group was followed from dawn to dusk, with the help of a field assistant, and tool use (as defined by Shumaker, Walkup, and Beck)^29^ episodes were recorded by “All Occurrences” sampling^30^. Tool use is infrequent when compared to other behaviors from capuchin monkeys’ repertoire, making “Focal Sampling” an inadequate method when trying to register tool use, so we choose the “All Occurrences” sampling as a more viable method, even if it makes the individual analysis more difficult.

Tool use behaviors were registered by voice and/or by video. The monkey (or its sex and age group when individual identification was not possible), and the target were identified when possible. We used the following age categories: infant (0–2 years), juvenile (2–5 years), subadult (only males, 5–7 years), and adult (more than 7 years or 1^st^ pregnancy). Most ages were estimated at the beginning of data collection, because the groups were not previously studied.

The tool use rate was calculated by summing up all tool use episodes observed in a month and dividing it by the number of contact hours with that group and by the number of individuals in the group that month, so this data is presented in the form “episodes/h/individual”.

The stone tools were collected after the monkey left the excavation site, whenever we were sure about the stone actually used by the subject. We measured the weight, length, width and thickness of the collected tools (for details on the measurement methodology, see Falótico and Ottoni)^1^.

### Meteorological data

The rainfall data were provided by the Instituto Nacional de Meteorologia (INMET), collected from the São Raimundo Nonato automatic meteorological station^31^ located about 27 km from the research area. This was the closest meteorological station at the time of the research.

### Food availability

For the measurement of food availability, we used a transect covering the three topographies of the area (plain, valley and mesa). It comprised 130 points total (26 in plain, 7 in valley and 97 in mesa; 30–40 m apart), each of them containing one plant matter collector (345 cm^2^ of area) and one pitfall trap (78.5 cm^2^ of area). The three environments were sampled according to their representative cover of the park area, in an area 5 km from the groups home ranges, to avoid tourist visiting areas. We retrieved the material monthly, drying, weighting and identifying the plant matter (flowers, fruits and seeds) from the collectors, and the arthropods from the traps. These data are presented in g/m^2^.

We also collected, by digging, some of the trapdoor spiders in the area, to identify the species the monkeys could be preying on. Because we could not get the actual spiders the monkeys captured, we are assuming they were predating some of the species we collected.

### Statistical analyses

Because of the nature of the data on tool use, i.e., low frequency and longitudinal repeated measures, we applied Generalized Linear Mixed Models (GLMM) to control for unequal repetition of the same individuals over time when analyzing the individual tool use frequencies^32^. We used a GLMM model to test the effect of the following independent variables on dependent variable tool use counts: rain precipitation, arthropod, and plant matter production per area. The Poisson distribution for dependent variables was used to correct for the excess of zeroes on the tables, the logarithmic link function was used, with individuals as a random effect, and logarithm of time of contact was used as offset variable in order to normalize the dependent variable.

To compare food availability between dry and rainy seasons, we used a GLMM model to test the effect of the independent variable rain precipitation (fixed effect) on each target dependent variable (arthropod and plant) matter availability on. A gamma distribution for dependent variable was used, with a log link function. The topography area (plain, valley and mesa) of the collectors was used as random effect.

We used a GLMM model to test the main effect of the independent variables sex, age class, and resource (louro, spider, USOs), and interaction effect sex-age on dependent variable stone tool use success (the frequency of the stone tool use events in which the resource was effectively obtained). A binomial distribution for dependent variable was used, with the logit link function, and with individual and group as random effects.

To compare the dimensions of the stone tools between groups, sex, ages classes we used GLMM model to test the effect of the independent variables sex, age class and resource on dependent variables stone tool size and weight. Normal distribution for dependent variable was used, with the identity link function, and with individual and group as random effect. Resource was used as a fixed control effect.

All tests were analyzed with a 0.05 significance level with Bonferroni correction, and all analyses were performed in IBM SPSS Statistics 23.

### Ethical Note

The research in SCNP was previously approved by IBAMA/ICMBio (authorizations 037/2007 and 14825-1), adhered to the American Society of Primatologists principles for the ethical treatment of primates, and followed all ethical guidelines for animal research of the Institute of Psychology-USP.

### Data Availability

The datasets generated during and/or analyzed during the current study are available from the corresponding author on reasonable request.

## Results

The groups were systematically followed for 20 days per month, from initial visual contact in the morning until the end of the day or the loss of contact with the group. The data from PF group were collected for 23 months (Sep 2007–Jul 2009 - total contact time of 1290.23 h), and from BC group, for 12 months (Mar 2008–Feb 2009 - total contact time of 426.36 h).

**Figure 1:**
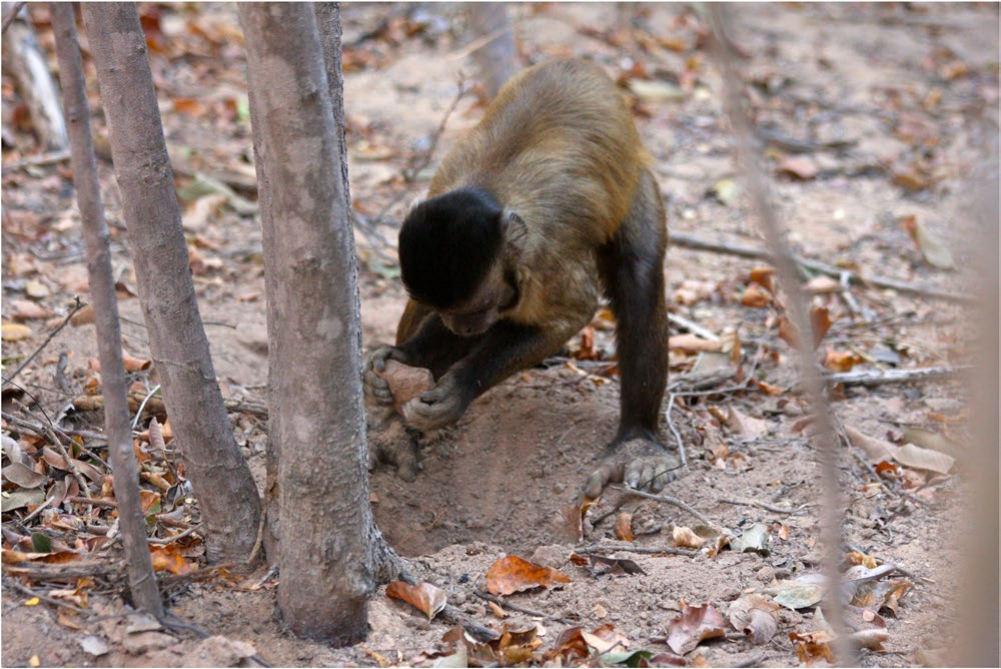
Male capuchin monkey digging with a DST.

We registered 1702 episodes of digging with stone tools by monkeys of the two groups (Fig. 1; Supplementary Video S1); the rate of occurrence of stone-aided digging was 0.0125 ep/h/indiv. Most (95%) adults/subadults individuals from both groups (N = 44) were registered using stone tools for digging during this study (see Supplementary Table S2 for individual frequencies). The typical use of the stones was as Digging Stone Tools (DST) to loosen the soil, using the stone tool as a percutor to do so; however, some stones were also used to pull the dirt from the digging site, as “hoes”. The latter was classified as a subcategory (DSTh).

**Figure 2:**
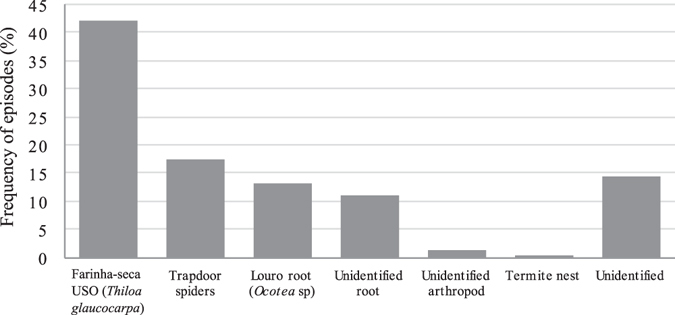
Food items searched by the monkeys with the aid of DSTs. *Unidentified* resources correspond to episodes in which we were unable to determine the target resource even after site inspection.

Several food items were targeted by the monkeys when digging (Fig. 2). Digging could be performed in a previously dug site or in an unexplored site. When the monkeys were looking for roots, they frequently re-used sites previously dug not only by monkeys, but also by collared peccaries (*Pecari tajacu*) that consume some of the same roots (TF, personal observation).

**Figure 3:**
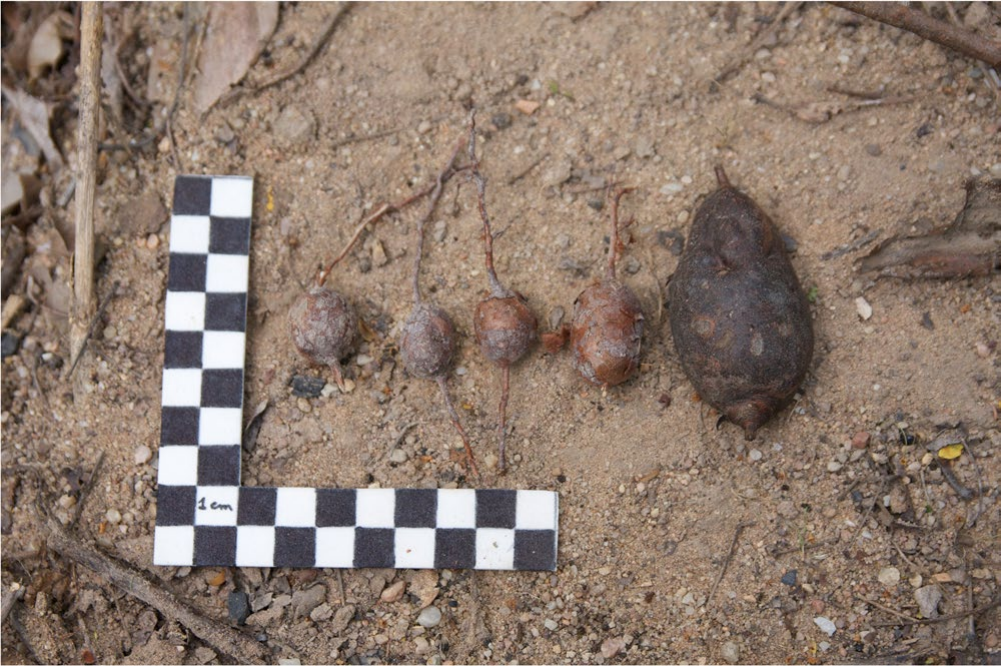
*Thiloa glaucocarpa* underground storage organs (USOs). Scale: 10 cm.

The most frequently dug up resources were *farinha*-*seca* USOs (*Thiloa glaucocarpa* – Fam. Combretaceae). These USOs can reach 10 cm length (Fig. 3) and are rich in carbohydrates. The roots did not appear to be consumed, so the target was the tuber. Although the leaves of this species are toxic to cattle, containing tannins and saponins^33^, the roots do not seem to be toxic to the monkeys.

The consumption of the USOs after excavation also required the removal of the external fibrous hard layer, which was usually done with the hands and teeth. However, in some occasions, the monkeys used stones to smash the USOs and thus access the inner edible part, especially if the USO was large^1^.

**Figure 4:**
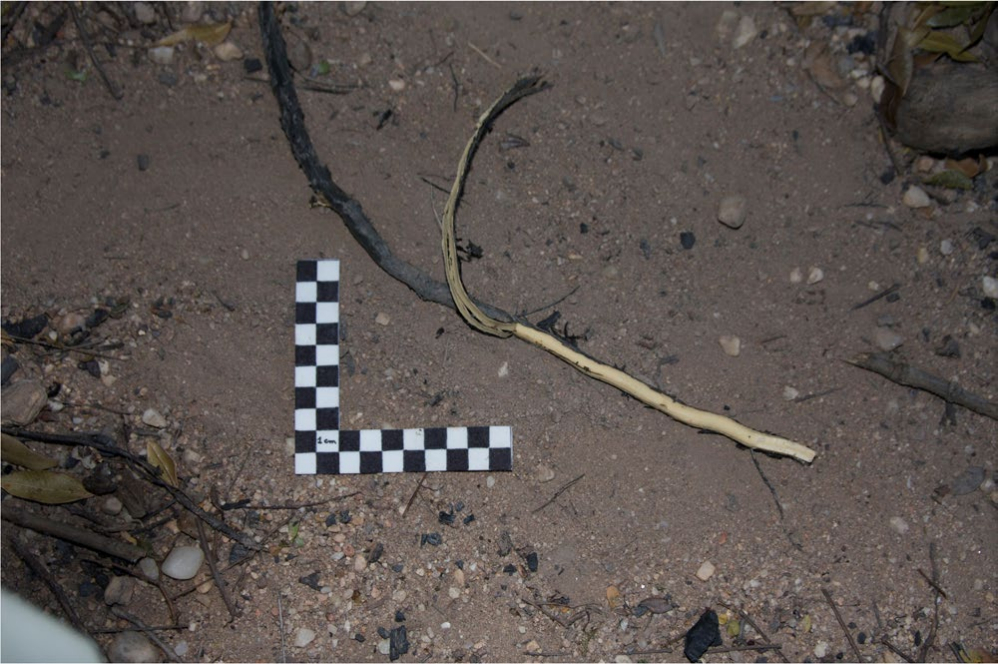
Louro tree root (*Ocotea* sp), with peeled skin. Scale: 10 cm.

Another widely used resource were the roots of the *louro* tree (*Ocotea* sp - Fam. Lauraceae), dug up beside the trunk of the tree (Fig. 4). The monkeys peel the dark-skinned roots before the consumption of their cores, usually by rubbing the roots between the hands or against trunks or rocks.

**Figure 5:**
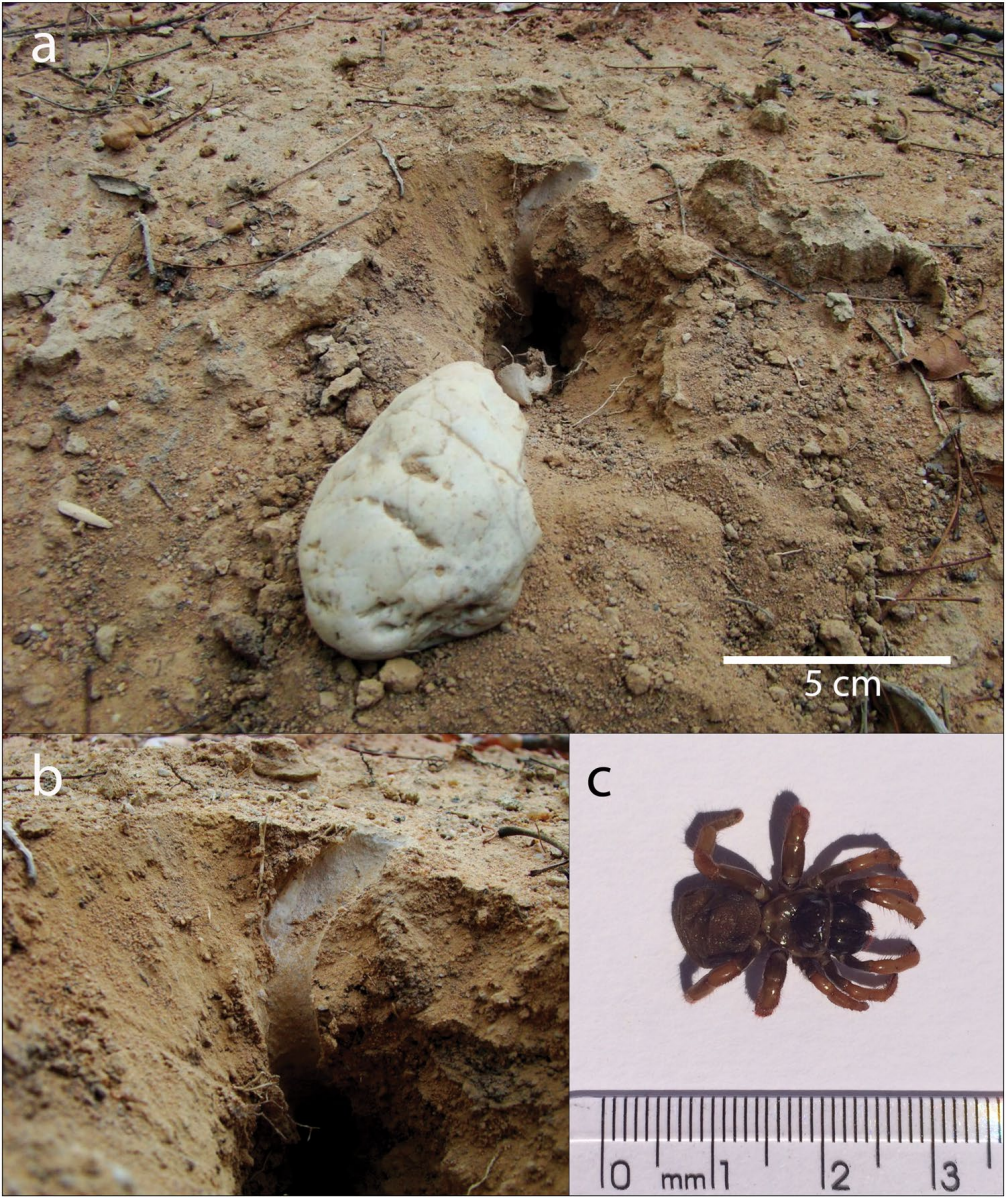
Capuchin monkey digging site. (**a**) Excavated tunnel and the DST used. (**b**) Detail of the web tunnel. (**c**) Trapdoor spider (*Actinopus* sp).

The most excavated resource besides plants’ USOs were trapdoor spiders (e.g. *Actinopus* sp – family Actinopodidae; *Magula* sp – family Theraphosidae; unknown spp – family Nemesiidae). These spiders build web-coated tunnels and cover the entrance with a camouflaged web cap (Fig. 5). While foraging on the ground, monkeys find the web cap, identifying the tunnel and initiating the excavation (Supplementary Videos S3 and S4).

**Figure 6:**
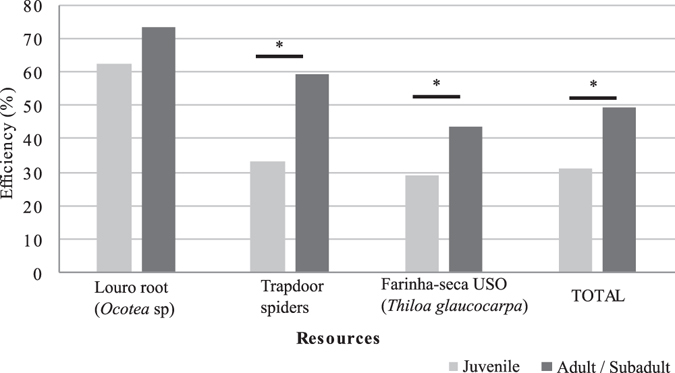
Efficiency in the acquisition of resources using DSTs. Only episodes with resource identification are accounted. (n juveniles = 628; n adults/subadults = 613). * GLMM, p < 0.05.

The overall success rate in tool use episodes (acquisition and consumption of the resource/n events) was 38%, and when the episodes were classified by age and resource, there were significant differences (Fig. 6).

The juveniles had a lower overall success rate than the adults (GLMM, F 4.903, df1 = 1, df2 = 636, p = 0.027). Although similar in performance to adults when digging for roots, juveniles performed much worse when trying to dig spiders and USOs (Fig. 6).

DST use by males was much more frequent (77%, N = 1135) than by females (23%, N = 429). There was no difference, though, between the success rates of each sex (GLMM, F 0.632, df1 = 1, df2 = 636, p = 0.427).

We collected and measured 703 stone tools (Table 1). Most were used only as DSTs, but 25 were also used as DSTh (to pull the loosened soil).

**Table 1:** Averages of the DSTs measurements.

	Mean
Weight	126.1 ± SD 124.8 mm
Length	6.2 ± SD 2.0 mm
Width	4.0 ± SD 1.3 mm
Thickness	3.0 ± SD 1.0 mm

Tools used by both groups, N = 703.

There was a sex difference in the size of the DSTs, males using heavier and larger tools (Tables 2 and 3). However, there was no difference in the efficiency of the tools based on weight (GLMM, F = 0.005, df1 = 1, df2 = 299, P = 0.945).

**Table 2:** DSTs’ size and weight by sex (n male used tools = 561; n female used tools = 165).

	Sex	Mean
Weight	Male	146.49 ± SD 148.93 g
	Female	106.20 ± SD 114.22 g
Length	Male	6.66 ± SD 2.83 mm
	Female	6.12 ± SD 3.43 mm
Width	Male	4.16 ± SD 1.28 mm
	Female	3.86 ± SD 1.11 mm
Thickness	Male	3.10 ± SD 1.10 mm
	Female	2.84 ± SD 0.91 mm

**Table 3:** GLMM results for the stone dimensions (weight, length, width and thickness) and the predictor variables group, sex and age (juveniles and adults/subadults), N = 301.

	*Effect*	*B*	*SE*	*F*	*df1*	*df2*	*p*-*value*
*Weight*	Intercept	55.616	41.749	4.599	3	297	0.004
	Group	−3.610	25.047	0.021	1	297	0.886
	Age	81.621	32.010	6.502	1	297	0.011
	Sex	47.551	21.267	4.999	1	297	0.026
*Length*	Intercept	5.139	0.534	5.914	3	291	0.001
	Group	0.017	0.322	0.003	1	291	0.958
	Age	1.084	0.408	7.054	1	291	0.008
	Sex	0.776	0.273	8.100	1	291	0.005
*Width*	Intercept	3.396	0.321	6.559	3	291	0.001
	Group	−0.110	0.194	0.323	1	291	0.570
	Age	0.781	0.245	10.129	1	291	0.002
	Sex	0.382	0.164	5.439	1	291	0.020
*Thickness*	Intercept	2.289	0.272	7.146	2	291	0.001
	Group	0.027	0.164	0.027	1	291	0.869
	Age	0.706	0.208	11.498	1	291	0.001
	Sex	0.370	0.139	7.071	1	291	0.008

There were differences in the physical properties of the tools according to the targets (GLMM, F = 12.606, df1 = 2, df2 = 241, P < 0.001); the stones used for digging louro roots were heavier than the others (Table 4).

**Table 4:** Stone tool weight mean for each resource explored. N = 247.

	*Weight*	*N*
*Louro root*	283.0 ± SD 43.5 g	20
*Farinha seca USO*	129.7 ± SD 24.4 g	119
*Spider*	84.7 ± SD 27.0 g	108

There were significant differences in size and weight between the DSTs used by juveniles and by adults/subadults (Table 5), the latter using heavier and larger tools. Adults/subadults were also more efficient using these tools (47.5% success rate) than juveniles (32.5%) - GLMM, F = 5.740, df1 = 1, df2 = 832, P = 0.017.

**Table 5:** DSTs’ size and weight by age (n juveniles = 416; n adults/ subadults = 379).

	Age	Mean
Weight	Juvenile	107.63 ± SD 102.25 mm
	Adult/Subadult	163.18 ± SD 170.23 mm
Length	Juvenile	6.20 ± SD 3.45 mm
	Adult/Subadult	6.74 ± SD 2.16 mm
Width	Juvenile	3.82 ± SD 1.16 mm
	Adult/Subadult	4.31 ± SD 1.28 mm
Thickness	Juvenile	2.81 ± SD 0.90 mm
	Adult/Subadult	3.22 ± SD 1.15 mm

### Tools with Multiple Functions

We could identify 43 events (2.52% from total) in which the DSTs were also used as “hoes” (DSTh) to pull the dirt from the digging site. This behavior was observed in both groups (PF- 31, BC-12) and by both sexes (Males-36, Females-5, Unknown-2), albeit with a great male bias.

### Group comparisons

Compared to the other SCNP capuchin groups studied before^7, 21^, PF/BC groups used stone tools for digging with similar frequency of that reported by Mannu and Ottoni^7^, 0.013 ep/h/indiv versus 0.010 ep/h/indiv; and less frequently than reported by Moura and Lee^21^, 0.028 ep/h/indiv. PF/BC groups had a higher overall success rate than the groups studied by Mannu and Ottoni (38% versus 21.6%), and a similar rate to that reported by Moura and Lee (40.8%).

### Food availability

**Figure 7:**
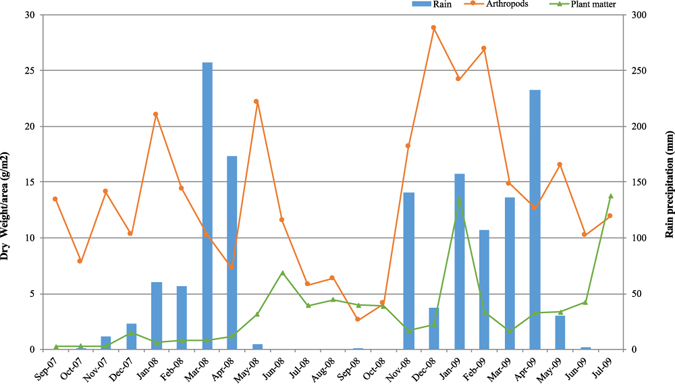
Arthropods availability (orange line), plant matter availability (green line), and rain precipitation (bars) during the research period.

The arthropods and plant matter collection provided indirect measures of the monthly food availability in the area (Fig. 7). The correlation between rain precipitation and resource abundance was significant, but very weak, for both arthropods (GLMM, F = 4.504, df1 = 1, df2 = 66, B = −0.002, p = 0.038) and plant matter (GLMM, F = 30.890, df1 = 1, df2 = 64, B = 0.006, p < 0.001). The correlation was negative for arthropods and positive for plant matter.

The GLMM to test the main effects of tool use rate, food availability and rain precipitation, involved a regression of the individual monthly tool use rate, based on a Poisson distribution (Table 6). The only significant main effect was the availability of plant matter. Moreover, the effect was positive, meaning that stone tool use for digging happened more frequently when more plant matter were available. The rain was marginally significant, but with a very weak negative effect.

**Table 6:** Results for the Generalized Linear Mixed Model (GLMM) for model effects on the response tool use count by individual per month and predictor variables *food availability* and *rain precipitation*.

*Effect*	*B*	*SE*	*F*	*df1*	*df2*	*p*-*value*
*Intercept*	−4.035	0.154	29.028	3	643	0.000
*Plant matter*	0.109	0.013	74.974	1	643	0.000
*Arthropods*	−0.009	0.013	0.478	1	643	0.490
*Rain*	−0.003	0.001	3.699	1	643	0.055

We used the log of contact time with the individual group as offset variable.

## Discussion

Digging for USOs and other buried resources with aid of tools is customary (sensu Whiten *et al*.)^34^ in all groups studied in SCNP. So far, this is the only known capuchin monkey population where the use of digging tools is a customary behavior.

There were differences in the size of the tools used by juveniles and adults, and by males and females, probably because of their different body sizes, but these differences did not seem to affect the use of the tools. The stone tools used to dig *Ocotea* roots were heavier and thicker - which could be a consequence of a different soil (e.g. more compact) around those trees (soil texture was not measured, though).

Despite the digging technique being similar for all resources, some resources are harder to locate than others, probably requiring more experience to be exploited, which could explain the higher success rates of adults, as compared to juveniles’, for some kinds of resources. The *Ocotea* roots (for which the success rates have little difference between these age groups) are very easy to locate and can be dug from already used sites, because this resource (the root) is not quickly exhausted (as are discrete items like spiders or USOs).

The detection of spiders and USOs seems much more difficult. Trapdoor spiders are solitary - there is only one per tunnel (once the prey is eaten the site is depleted). Moreover, the monkeys must learn to identify the web cap of an intact tunnel: juveniles were occasionally seen digging up spider tunnels already used, a behavior not observed in adults (T. Falótico, personal observation).

The same is somehow true for *Thiloa* USOs, which are not present in every root, making the search more challenging than *Ocotea* roots. On the other hand, these USOs are also dug and eaten by peccaries, so sites dug by them could be used as a hint of USOs locations. These characteristics appear to make spiders the most difficult resource to locate, followed by the USOs, which relates to the different success rates of juveniles and adults. Roots and USOs have been considered fallback food for capuchin monkeys in this area^22^, but the present data do not support this claim.

The use of stones as “hoes” (DSTh) was infrequent, as compared to that of the DSTs used only as “hammers”. Only 14 individuals out of 61 performed both behaviors. Individuals in both groups used stones as DSThs, and although we have registered a small number of events, it appears to be habitual in the groups.

The difference in the frequency of digging stone tool use between the groups studied in SCNP could be due to environmental or seasonal differences, or to missing observations of episodes happening at the same ti﻿me, a sampling issue in larger groups. PF and BC groups (this study) and Jurubeba group^7^ were 3 to 4 times larger than the 10 individuals group reported by Moura and Lee^21^, making missing observations of episodes happening at the same time more likely for the larger groups. Ecology is not a likely explanation (although we cannot discard it), since both areas are just 15 km apart, but inter-annual seasonality could account for some variation in the overall availability of food items obtained using stone tools, since data were collected in different years, and this region has great inter-annual rain variation^31^, a factor that could affect plant productivity.

The difference in success rate of DST use could be due to distinct inter-annual availability of the more easily obtained underground food items, such as roots (as compared to USOs or spiders). Unfortunately, neither of those previous studies presented data separated by resources consumed. Although we could not compare the food consumption frequencies for each resource with those studies, some of the consumed items are the same, like *T*. *glaucocarpa* ^23^ while others were not observed being consumed in the present study - *Combretum* sp and *Astronium* sp^7^. Both previous studies reported digging of insects’ nests, but there is no mention of spiders. Again, this could be due to a difference in their availability between the areas or to the misidentification of spider tunnels as insect´s nests.

We found no negative correlations between plant and arthropods’ availability and digging tools’ use frequency, as predicted by the “necessity hypothesis”. On the contrary, we found a significant positive correlation between DST use and plant matter availability, similarly to what was found for the nut cracking tools’ use in another *S*. *libidinosus* population^28^, making the lack of preferred food an unlikely factor to predict the occurrence of stone tools’ use. These results are also similar to those on chimpanzees’ USOs consumption and tool use^14^. For capuchin nut cracking, the nuts were considered a staple fallback food^28^, while the present underground resources would be more precisely described as a filler fallback food (a resource that never fills up the whole diet, is not consumed during part of the season and is patchily distributed). An alternative, non-exclusive, hypothesis tested and supported in other population studied - but not, so far, in SCNP - is the “opportunity hypothesis”^28^. It proposes that tool use to access encased food is invented and maintained by repeated exposure to appropriate ecological conditions, such as the presence of the resource that needs tools to be consumed and the tool materials^28, 35^.

The use of stone tools for digging is present in all the studied groups in the SCNP population. Its apparent absence in other tool-using capuchin monkey populations^36^ could be explained by distinct social traditions, since ecological and genetic differences are apparently small. The “opportunity hypotheses” could also explain the situation, because both the resource to be processed *and* the adequate lithic material have to be available. The abundance of stone material, high in SCNP, could facilitate the innovative use of stones as tools for several purposes^1, 7, 24, 25, 37^.

The hypothesis could be used as a complementary explanation for the variation of stone tool use or any object manipulation behaviors in other non-human primates. Groups that use the same kind of object but live in areas with different availability of proper material would present a positively correlated variation in tool use diversity.

## Electronic supplementary material

Supplementary Video S1

Supplementary Table S2

Supplementary Video S3

Supplementary Video S4
