# Supplementary material for: Digging up food: excavation stone tool use by wild capuchin monkeys
Source: Sci Rep. 2017 Jul 24;7:6278. doi: 10.1038/s41598-017-06541-0 (PMC5524703; doi:10.1038/s41598-017-06541-0)
Supplement: Supplementary file 2 — Supplementary Table S2 [file 41598_2017_6541_MOESM2_ESM.doc]

**Digging up food: excavation stone tool use by wild capuchin monkeys**

Tiago Falótico, José O. Siqueira, Eduardo B. Ottoni

Suplemmentary Table S2 – Individual frequency of digging tool use for each individual in Pedra furada (PF) and Bocão (BC) groups from Sept/2007 to Jul/2009

| **Subject** | **Age** | **Sex** | **Group** | **Frequency** |
| --- | --- | --- | --- | --- |
| Bacana | Adult | M | BC | 6 |
| Berinjela | Juvenile | M | BC | 1 |
| Bocão | Adult | M | BC | 11 |
| Bruxa | Adult | F | BC | 2 |
| Capenga | Subadult | M | BC | 17 |
| Cassia | Adult | F | BC | 1 |
| Cheng | Juvenile | M | BC | 7 |
| Diana | Adult | F | BC | 7 |
| Doente | Juvenile | M | BC | 1 |
| Elvira | Adult | F | BC | 2 |
| Lola | Adult | F | BC | 3 |
| Perninha | Adult | F | BC | 2 |
| Porthos | Adult | M | BC | 16 |
| Rabugento | Adult | M | BC | 23 |
| Sophia | Adult | F | BC | 5 |
| Tara | Adult | F | BC | 1 |
| Willy | Adult | M | BC | 12 |
| Ajudante | Juvenile | F | PF | 4 |
| Alice | Juvenile | F | PF | 4 |
| Anne | Adult | F | PF | 2 |
| Apolo | Adult | M | PF | 12 |
| Assustado | Subadult | M | PF | 9 |
| Beiçola | Adult | M | PF | 22 |
| Benne | Adult | F | PF | 21 |
| Blip | Juvenile | M | PF | 30 |
| Blue | Juvenile | M | PF | 27 |
| Bochecha | Subadult | M | PF | 3 |
| Bochechudo | Adult | M | PF | 17 |
| Canela | Adult | F | PF | 35 |
| Caveira | Adult | M | PF | 18 |
| Chifrudo | Juvenile | M | PF | 51 |
| Encrenqueira | Adult | F | PF | 26 |
| Falsa | Adult | F | PF | 2 |
| Gorda | Adult | F | PF | 18 |
| Igor | Subadult | M | PF | 20 |
| Imigrante | Subadult | M | PF | 1 |
| Jurema | Adult | F | PF | 12 |
| Lica | Adult | F | PF | 33 |
| Limão | Juvenile | M | PF | 20 |
| Maçã | Adult | F | PF | 15 |
| Manco | Adult | M | PF | 7 |
| Marronzinho | Juvenile | M | PF | 5 |
| Mercurio | Juvenile | M | PF | 1 |
| Molenga | Adult | M | PF | 13 |
| Nemo | Infant | M | PF | 6 |
| Nico | Subadult | M | PF | 64 |
| Ninfa | Adult | F | PF | 5 |
| Orelha | Adult | M | PF | 8 |
| Pedrita | Adult | F | PF | 3 |
| Pretinho | Juvenile | M | PF | 43 |
| Rabo Pelado | Juvenile | M | PF | 4 |
| Ramela | Juvenile | F | PF | 11 |
| Roger | Subadult | M | PF | 13 |
| Romã | Adult | F | PF | 17 |
| Saco de Pancada | Juvenile | M | PF | 18 |
| Sapata | Adult | F | PF | 1 |
| Songo | Juvenile | M | PF | 4 |
| Tatu | Adult | F | PF | 3 |
| Torto | Adult | M | PF | 33 |
| Vesga | Juvenile | F | PF | 38 |
| Zandor | Adult | M | PF | 22 |
| No identified | - | - | - | 864 |
| **Total** |  |  |  | **1702** |
